# Dimer Formation Enhances Structural Differences between Amyloid β-Protein (1–40) and (1–42): An Explicit-Solvent Molecular Dynamics Study

**DOI:** 10.1371/journal.pone.0034345

**Published:** 2012-04-11

**Authors:** Bogdan Barz, Brigita Urbanc

**Affiliations:** Physics Department, Drexel University, Philadelphia, Pennsylvania, United States of America; University of Akron, United States of America

## Abstract

Amyloid 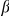-protein (A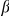) is central to the pathology of Alzheimer's disease. A 5% difference in the primary structure of the two predominant alloforms, A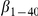 and A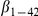, results in distinct assembly pathways and toxicity properties. Discrete molecular dynamics (DMD) studies of A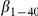 and A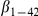 assembly resulted in alloform-specific oligomer size distributions consistent with experimental findings. Here, a large ensemble of DMD–derived A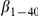 and A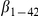 monomers and dimers was subjected to fully atomistic molecular dynamics (MD) simulations using the OPLS-AA force field combined with two water models, SPCE and TIP3P. The resulting all-atom conformations were slightly larger, less compact, had similar turn and lower 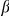-strand propensities than those predicted by DMD. Fully atomistic A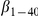 and A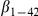 monomers populated qualitatively similar free energy landscapes. In contrast, the free energy landscape of A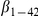 dimers indicated a larger conformational variability in comparison to that of A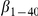 dimers. A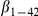 dimers were characterized by an increased flexibility in the N-terminal region D1-R5 and a larger solvent exposure of charged amino acids relative to A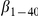 dimers. Of the three positively charged amino acids, R5 was the most and K16 the least involved in salt bridge formation. This result was independent of the water model, alloform, and assembly state. Overall, salt bridge propensities increased upon dimer formation. An exception was the salt bridge propensity of K28, which decreased upon formation of A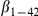 dimers and was significantly lower than in A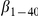 dimers. The potential relevance of the three positively charged amino acids in mediating the A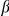 oligomer toxicity is discussed in the light of available experimental data.

## Introduction

Alzheimer's disease (AD) is the leading cause of dementia among the elderly. Substantial evidence implicates the amyloid 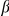-protein (A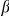) in triggering a cascade of events that eventually lead to neuronal loss. There are two dominant alloforms of A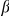 in the brain, A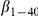 and A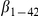. Both A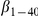 and A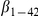 have a high propensity to assemble into soluble, quasi-spherical oligomeric assemblies and further form insoluble fibrils with a characteristic cross-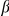 structure typically found in extracellular amyloid plaques in the AD brain. Genetic, pathologic, and biochemical evidence strongly supports the hypothesis that low-order oligomeric assemblies of A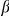, rather than fibrils, are the proximate neurotoxic agents in AD [1]–[7]. Despite a relatively small difference in the primary structure, with A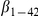 having additional two C-terminal residues I41-A42, A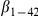 aggregates faster [8], [9], is genetically linked to aggressive, early-onset familial forms of AD [10], and is more toxic [5] than A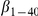 *in vitro* [11], [12] and *in vivo* [13], [14].

Experimental studies of A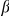 assembly pathways and structural characterization of resulting A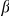 oligomers are critically limited by their transient and heterogeneous nature. A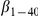 and A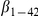 oligomer size distributions were characterized *in vitro* by photo-induced cross-linking of unmodified proteins (PICUP) combined with gel electrophoresis (SDS-PAGE) to demonstrate their distinct oligomerization pathways [15]. Whereas A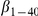 formed monomers through tetramers, in descending abundance order, A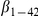 showed in addition an increased abundance of pentamers and hexamers that assembled further to form decamers to dodecamers [15]. Similar observations on distinct A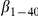 and A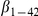 assembly pathways were later made by Bernstein *et al.* using ion mobility-mass spectrometry (IMS-MS) that does not require cross-linking chemistry [16]. Importantly, the assembly differences and the distinct toxicity properties originate in a relatively small difference (5%) between A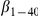 and A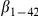 primary structures.

While a variety of biophysical experimental techniques provided a few glimpses into A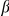 monomer and oligomer structures in aqueous solution, experimentally-derived three-dimensional A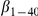 and A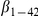 oligomers are not available. Numerous computational approaches have been applied to elucidate A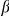 monomer and oligomer structures [17], [18]. An efficient discrete molecular dynamics (DMD) combined with a four-bead protein model with backbone hydrogen bonding and amino acid-specific interactions was applied to folding [19], [20] and oligomer formation of A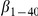, A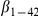, and their Arctic mutants [19], [21]. This DMD approach was shown to yield oligomer size distributions of all four full-length A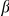 peptides [29] consistent with PICUP/SDS-PAGE data [15], [22] and ion mobility/mass spectroscopy results [16]. DMD-derived A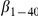 and A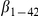 oligomers were quasi-spherical structures with hydrophobic regions comprising the core and hydrophilic regions located at the surface [19], [21]. The DMD approach predicted a turn centered at G37–G38 in the A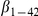 but not in the A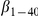 monomer structure [19]. This structural difference was later observed *in vitro* and confirmed *in silico* [23]–[27]. A rather unexpected structural difference between DMD-derived A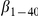 and A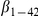 oligomers involved their N-terminal region D1–D7. A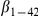 oligomers had substantially increased solvent exposure of the D1–D7 region relative to A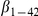 oligomers [19], [21], a feature that was recently observed by all-atom MD in A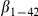 monomers [28]. Urbanc *et al.* hypothesized that this structural difference was critical for distinct toxicity properties of A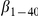 and A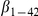 oligomers [19]. In a recent DMD study, this hypothesis was further corroborated by showing that the effective peptide inhibitors of A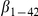 toxicity significantly decreased the solvent exposure of the N-terminal region D1–D7 of A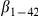, in contrast to the ineffective inhibitors [21].

The comparison of the structural predictions of the DMD approach to the *available* experimental data [19]–[21] demonstrated that the DMD approach is a powerful tool for elucidation of A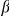 assembly pathways and structures. The question remains whether the DMD-derived structural differences between A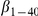 and A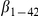 assemblies are an artifact of the DMD approach, which uses a coarse-grained protein structure and square-well potentials combined with an implicit solvent. Experimental characterization of N-terminal structural characteristics is complicated by the fact that the N-terminal region of full-length monomers and oligomers is the least structured region and thus more sensitive to solvent conditions and experimental probes. We here hypothesized that the DMD-derived A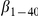 and A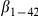 conformations are structurally similar to fully atomistic conformations, and selected a large ensemble of DMD-derived A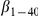 and A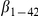 monomers and dimers as initial conformations for an all-atom MD study in explicit water. Our aim was to structurally compare fully atomistic A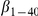 and A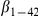 monomers and dimers, quantify their structural differences, and thereby elucidate those structural elements that may be associated with distinct toxicities of A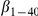 and A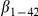 oligomers observed both *in vitro* and *in vivo*. A multiscale approach that combined coarse-grained modeling and all-atom MD, similar to ours, was recently shown by Samiotakis *et al.* to be even more efficient than all-atom REMD [30].

To select the force field and water model for our study, we examined the previous explicit solvent all-atom MD studies targeting folding of full-length A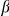 [26], [28], [31]–[46]. These studies largely differed by the choice of the force field, the solvent treatment (either implicit or explicit), and, in the case of explicit solvent, by the choice of the water model. Many MD studies used replica exchange MD (REMD) for a more efficient sampling of the conformational space. Among the explicit water models, TIP3P and SPCE were used most frequently, though recently, Sgourakis *et al.* [44] reported REMD simulations of A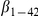 folding using the AMBER force field ff99SB combined with TIP4P-Ew water model that was previously applied to a REMD study of A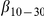 folding by Fawzi *et al.* [47]. The choice of a water model was recently shown to strongly influence the accuracy of hydration thermodynamic properties of amino acid analogues whereas the differences resulting from application of different force fields were smaller [48]. Among the non-polarizable water models combined with three most common biomolecular force fields, the SPCE model resulted in overall the best agreement with experimental data [48].

Several implicit solvent computational studies were also applied to characterize full-length A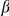 monomers and dimers [27], [43], [49]–[55]. Monomers of A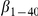, A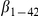, and a few selected mutants were studied by implicit solvent Monte Carlo simulations [51], A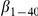 and/or A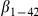 were examined by all-atom implicit solvent REMD [49], [50] and by coarse-grained implicit solvent REMD [27]. Dimers of A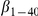 and A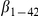 were examined by all-atom implicit solvent Monte Carlo simulations [55], whereas N-terminally truncated, A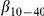 dimers were studied by implicit solvent REMD [52], [53]. The present study is unique as it combines the coarse-grained DMD approach with all-atom MD in explicit solvent to examine and compare a large ensemble of fully atomistic structures of both A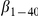 and A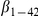 monomers and dimers aimed at characterizing structural changes involved in the first step of assembly from monomeric to dimeric states. By using two explicit water models, SPCE and TIP3P, we were able to examine in addition the robustness of the resulting structures with respect to the water model and to examine the effect of explicit protein-water interactions on the resulting dimer structures. We characterized all salt bridge propensities in monomers and dimers of both alloforms and identified those that were alloform-specific, thereby quantifying structural changes occurring during monomer to dimer conversion for both A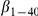 and A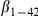 relevant to understanding A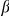-induced toxicity.

## Results

**Figure 1:**
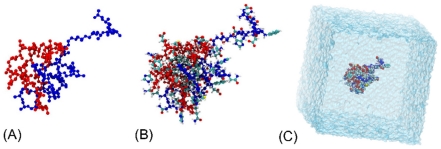
Reconstruction of fully atomistic A 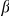
 **dimers from the DMD-derived four-bead A** 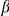 **dimer conformations.** *(A) A four-bead A* 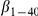 *dimer containing two peptides depicted in red and blue; (B) A fully atomistic dimer structure; (C) The all-atom dimer inserted in a cubic water box. Images were created by the VMD software package* [*90*] *.*

Dimer formation is the first step in the A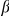 assembly into toxic oligomers. The purpose of this study was to quantify distinct structural properties of A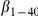 and A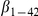 monomers and dimers using fully atomistic MD simulations in explicit water. MD simulations of full-length A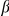 dimer formation are computationally demanding [17], [56]. We enhanced the sampling efficiency by using a large ensemble of different monomer and dimer structures of each A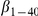 and A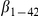, which were previously derived by the more computationally efficient DMD approach [21], as initial conformations in fully atomistic MD simulations in explicit solvent. The DMD-derived A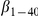 and A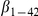 monomer and dimer conformations were converted into all-atom representations as described in the section *Methods* and illustrated in Fig. 1. The number of 50 ns long trajectories of A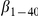 and A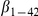 monomers and dimers acquired by all-atom MD using the SPCE and TIP3P water models is shown in Table 1. The structural results described below are based on 343 trajectories, each 50 ns long, that amounted to 17.15 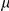
s of a total simulation time. No dissociation events in our all-atom MD dimer trajectories were observed for either water model. All acquired monomer and dimer trajectories were included into the analysis described below.

**Table 1:** The number of A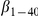 and A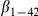 monomer and dimer trajectories.

	Monomers		Dimers	
	SPCE	TIP3P	SPCE	TIP3P
A 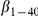	44 (26,400)	41 (24,600)	49 (29,400)	42 (25,200)
A 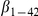	45 (27,000)	39 (23,400)	42 (25,200)	41 (24,600)

The number of different conformations used for structural analysis acquired between 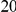 and 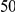 ns of each trajectory is given in parentheses.

In the following, we referred to the primary structure of A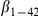:


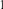
DAEFRHDSGY 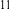
EVHHQKLVFF 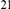
AEDVGSNKGA 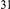
IIGLMVGGVV 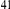
IA.

and A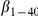, which is shorter by two C-terminal amino acids, I
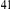
A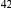.

### Convergence of A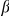 monomer and dimer trajectories

Because full-length A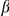 peptides are intrinsically disordered, sampling of the conformational space is an important aspect of any computational study that aims to characterize A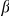 structures. As a measure of convergence, we monitored the root mean square distance (RMSD) values for all MD A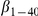 and A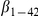 monomer and dimer trajectories. We selected five monomer and dimer trajectories with extreme RMSDs to show the lower and upper bounds for RMSDs of the entire ensemble of trajectories (Figs. S1 and S2). RMSD values converged within the initial 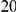 ns. In addition to RMSDs, we also monitored the time evolution of the average distance of the 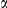 carbon atom of each amino acid from the center of mass (CM), hereafter referred to as the distance from the CM per amino acid, because it provided an intuitive measure of the structural arrangement of amino acids within monomers and dimers (Figs. S3 and S4). The convergence was reached within the first 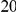 ns. We also tested the convergence of the distance from the CM in terms of the number of trajectories, which was equal to the number of initial DMD-derived conformers. These data demonstrated that the distance from the CM per amino acid converged for 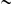
40 (or more) trajectories and that the convergence was faster for dimers than for monomers (Figs. S5 and S6). These results demonstrated that performing simulations for more than 20 ns and acquiring more than 40 different trajectories for each alloform, assembly state, and water model was critical for the distance from the CM per residue to converge.

Structural characterization described below was performed by considering conformations of all acquired trajectories for simulation times 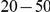 ns, resulting in at least 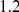 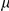
s of MD simulation time per conformational ensemble. For each quantity, described below, we calculated the average value and the standard error of the mean (SEM) using entire conformational ensembles. The structural differences between A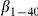 and A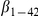 reported in this manuscript were based on those average quantities with non-overlapping SEM values.

### Conformational space sampled by A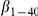 and A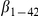 dimers

**Figure 2:**
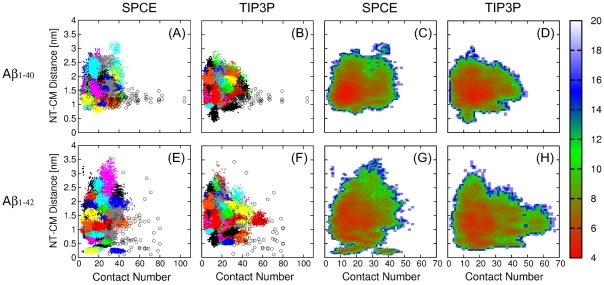
Sampling efficiency and free energy landscapes of A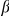 dimers. *Dimer conformations of all acquired MD trajectories of (A,B) A* 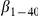 *and (E,F) A* 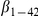 *, respectively, projected onto two reaction coordinates, for the (A,E) SPCE and (B,F) TIP3P water models. Each trajectory is shown in one color and each point corresponds to one dimer conformation along the trajectory acquired at simulation times 20–50 ns. The open black circles correspond to the initial DMD-derived dimer conformations. The PMF plots for (C,D) A* 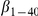 *and (G,H) A* 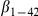 *dimers calculated from MD trajectories with the (C,G) SPCE and (D,H) TIP3P water model. The color scheme to the right of each plot is given in units of k* 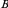 *T. Images were created by the GNUPLOT software package.*

To examine the conformational variability of A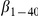 and A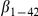 dimers, we constructed the PMF surface using the contact number and the distance of the N-terminal C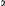 atom from the CM, the NT-CM distance, as reaction coordinates. The contact number, which is by definition the number of interpeptide contacts within a dimer, provided a measure of the contact surface area between the two peptides in a dimer. Because our results showed that the N-terminal amino acid D1 was the most solvent exposed amino acid in A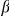 dimers, the NT-CM distance was used as an estimate of a dimer radius. The conformational sampling efficiency of the MD trajectories was estimated by projecting A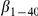 and A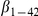 dimer conformations acquired at 20–50 ns onto the two reaction coordinates (Fig. 2). We noted a considerable overlap among conformations belonging to different MD trajectories. To facilitate a comparison to the DMD–derived initial dimer structures, Fig. 2 also shows the projections of the initial DMD dimer structures (open circles).

Comparing the DMD and all-atom MD conformations, we found that the DMD dimers were systematically shifted to larger contact numbers, indicating that the DMD approach overestimated the number of interpeptide contacts within dimers, resulting in more compact dimer structures. The effective radii (as measured by the NT-CM distances) of the DMD-derived A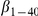 dimers were shifted to smaller values compared to the all-atom MD A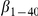 dimer radii (Fig. 2A and B). However, this shift was significantly smaller for A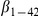 than for A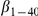 dimers (Fig. 2C and D).

The two water models resulted in slight yet systematic differences in the conformational space sampled by A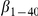 and A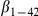 dimers. The SPCE water model resulted in a broader range of the NT-CM distances than the TIP3P water model, whereas the TIP3P water model yielded somewhat larger contact numbers, closer to those predicted by the DMD approach. A comparison of distributions of the two reaction coordinates, the contact number and the NT-CM distance, for A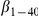 and A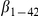 dimers showed significant alloform-specific differences (with non-overlapping SEMs) that depended on the water model, consistent with the above conclusions (data not shown).

The PMF minima of A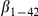 dimers were more dispersed and shallower than those of A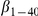 dimers, indicative of less stable A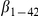 dimers compared to A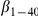 dimers. This interpretation is consistent with a notion that A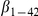 tends to assemble into larger oligomers than A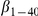 [15], [16]. All-atom MD A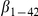 dimers sampled a broader region of the reaction coordinate space than A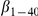 dimers for both water models, suggesting an increased variability of A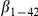 relative to A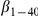 dimer structures. The free energy landscapes of A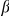 dimers were further explored and compared to monomer landscapes as described in the following.

### Distinct free energy landscapes of A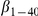 and A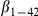 monomers and dimers

**Figure 3:**
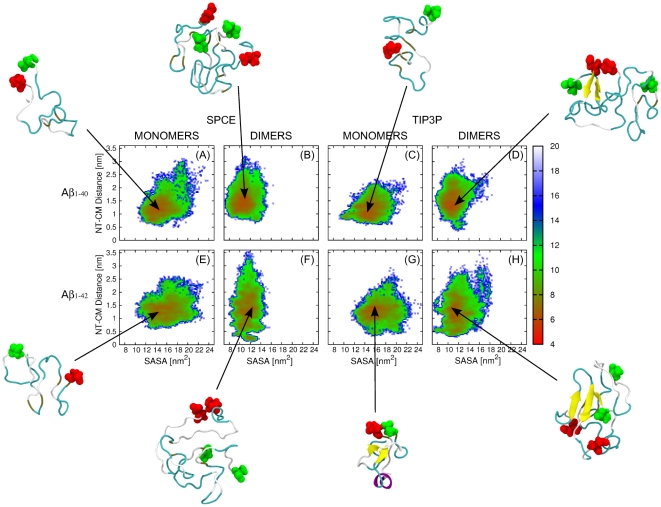
Free energy landscapes of A 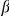
 **monomers and dimers with representative conformations.** *The reaction coordinates are the SASA of all hydrophobic amino-acids (x-axis) and the NT-CM distance (y-axis). The PMF plots for (A,C) A* 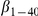 *and (E,G) A* 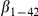 *monomers were acquired by MD using the (A,E) SPCE and (C,G) TIP3P water models. The corresponding PMF plots for (B,D) A* 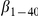 *and (F,H) A* 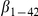 *dimers were acquired by MD using the (B,F) SPCE and (D,H) TIP3P water models. The color scheme to the right of each plot is given in units of k* 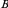 *T. The representative conformations of each conformational ensemble are displayed with the N-terminal amino acid D1 colored red and the C-terminal amino acid (V40/A42) colored green. The images were generated by VMD.*

We here examined A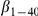 and A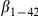 monomer structures to facilitate a comparison with dimers. To compare the free energy landscapes of A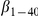 and A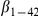 monomers and dimers, we calculated the PMF histograms using the NT-CM distance and the combined SASA of all hydrophobic residues as two reaction coordinates (Fig. 3). The NT-CM distance was selected as one of the two reaction coordinates because it discriminated the dimer structures of the two alloforms. The SASA of all hydrophobic residues was chosen based on an observation that the solvent exposed hydrophobic amino acids were critically involved in A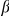 monomer to dimer conversion. In Fig. 3, representative structures of different conformational ensembles of A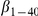 and A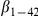 monomers and dimers are shown. These structures were identified as following. First, the conformations with the lowest PMF value were selected (84–197 per ensemble). Second, the resulting structures were clustered based on their pairwise RMSD values (with a cutoff of 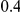 nm), as implemented within the GROMOS algorithm within the GROMACS software package. Third, the centroid of the largest resulting cluster was identified as a representative conformation. Although these structures provide a visual representation of A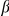 monomer and dimer conformations, representative monomer and dimer conformations of intrinsically disordered proteins do not provide a meaningful description of the entire conformational ensemble, as also concluded by other all-atom MD studies [46] and thus cannot serve as a substitute for a comprehensive structural analysis.

**Figure 4:**
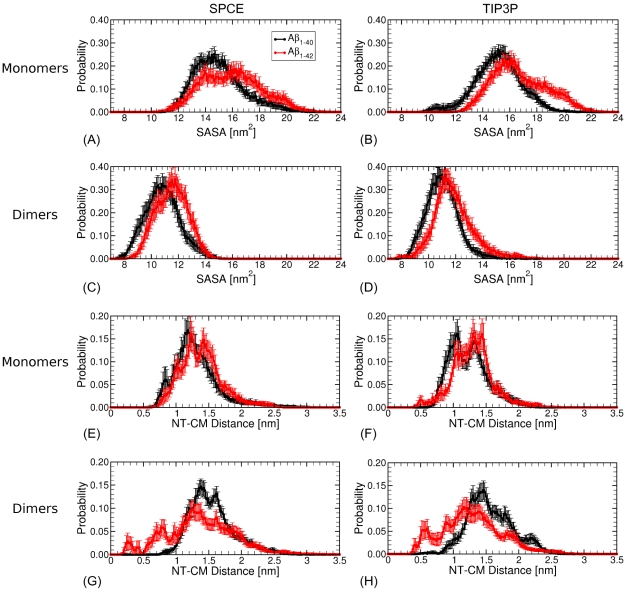
Probability distributions of SASA values and NT-CM values in MD-derived fully atomistic A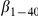 and A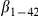 monomers and dimers. *The SASA value was calculated as a sum of SASA values over all hydrophobic residues for each monomer and dimer conformation. Similarly, the NT-CM distance was calculated for each monomer and dimer conformation. The resulting histograms were normalized to obtain probability distributions, displayed as black curves for A* 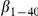 *and red curves for A* 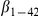 *monomers and dimers for each of the two water models. The error bars represent SEM values.*

As anticipated, we observed a significant shift of the free energy landscape toward lower SASA values upon monomer (Fig. 3A,E,C,G) to dimer (Fig. 3B,D,F,H) conversion for both A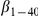 and A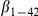 structures and for both water models. Fig. 4A–D shows normalized distributions of SASA values for monomers and dimers of both alloforms and for both water models. In the following, we calculated the average SASA and the corresponding SEM values. A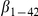 monomers had a larger average value of SASA of all hydrophobic residues (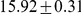 nm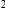 for SPCE and 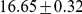 nm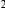 for TIP3P) than A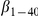 monomers (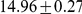 nm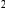 for SPCE and 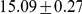 nm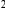 for TIP3P). A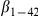 dimers also had a larger average value of SASA of all hydrophobic residues (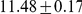 nm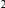 for SPCE and 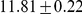 nm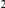 for TIP3P) than A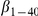 dimers (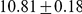 nm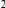 for SPCE and 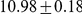 nm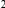 for TIP3P). This result is consistent with a view that oligomer formation is driven by a hydrophobic collapse, during which hydrophobic residues get effectively shielded from the solvent. Our data showed that this shielding was more efficient in A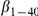 monomers and dimers that lack the two additional hydrophobic residues at the C-terminus of each peptide. A larger solvent exposure of hydrophobic residues in A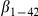 relative to A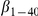 monomers and dimers might explain the larger aggregation propensity in the former.

A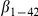 dimers populated a broader range of the NT-CM distances (Fig. 3F,H) than A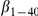 dimers (Fig. 3B,D), indicating a more flexible and less structured N-terminal region in A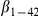 relative to A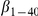 dimers (see A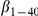 and A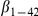 dimer dynamics displayed as Movie S1 and Movie S2). This result was observed for both water models but was more pronounced for the SPCE water model. We asked whether this structural difference between A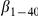 and A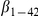 dimers was present also in monomeric states. Interestingly, in the SPCE water model, the A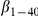 monomers displayed a slightly larger variability of the NT-CM distances than the A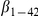 monomers (Fig. 3A,E), whereas in the TIP3P water model, the reverse effect was observed (Fig. 3C,G). Fig. 4E–H shows normalized distributions of NT-CM distances for monomers and dimers of both alloforms and for both water models. Overall, the structural differences between A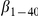 and A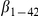 monomers were smaller than those between A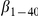 and A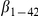 dimers, demonstrating that dimer formation enhances the initial structural differences between the two alloforms, with increased flexibility of the N-terminal region in A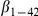 relative to A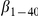 dimers, as predicted by the DMD approach [19], [21].

We examined the radius of gyration 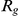 of all all-atom MD–derived A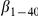 and A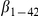 monomer and dimer conformations. The resulting average and standard deviations 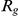 values were: 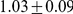 nm (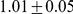 nm) for A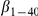 monomers, 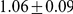 nm (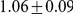 nm) for A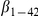 monomers, 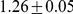 nm (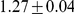 nm) for A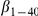 dimers, and 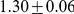 nm (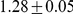 nm) for A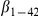 dimers obtained for the SPCE (TIP3P) water model, respectively. Recently, Ball *et al.* examined the ensemble of A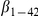 monomers by all-atom REMD in explicit solvent [45] and reported mostly compact although heterogeneous monomer conformations (90%) with 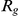 values that matched well with our present data [45].

Next, we examined the complexity of the free energy landscapes in terms of the number of minima and their depths. Upon A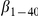 monomer to dimer conversion, the number of minima on the free energy landscape did not change. A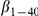 dimers were characterized by a slightly more compact free energy landscape and deeper minima than A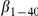 monomers. A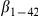 dimers had less compact free energy landscapes than A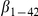 monomers in both water models. Importantly, the complexity of the free energy landscape *increased* upon A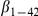 monomer to dimer conversion, suggesting that A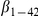 dimer formation resulted in a larger number of less stable dimer structures relative to A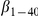 dimer formation.

### A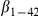 forms more 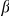-strand structure in the C-terminal region than A
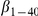


The secondary structure of A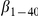 and A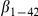 monomers and dimers mostly consisted of turns and 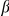-strands, and much less helical structure. The average percentages of the turn, 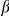-strand, helical, and coil content in A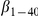 and A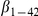 monomers and dimers are reported in Tables 2 and 3. The turn propensities per amino acid in MD-derived A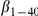 and A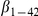 monomers and dimers were of the same magnitude as the turn propensities of the corresponding DMD monomers and dimers (Fig. S7). On the other hand, all all-atom MD conformations had on average lower 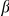-strand propensities than the corresponding DMD conformations. Fully atomistic A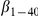 and A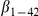 dimers did not show an increased 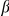-strand content relative to A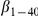 and A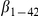 monomers. In contrast, DMD-derived dimers had a significantly increased 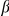-strand content relative to DMD-monomers, in agreement with experimental findings on cross-linked A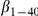 conformations [57].

**Table 2:** Average turn, 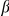-strand, helical, and coil propensities within A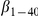 and A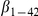 monomers obtained by the all-atom MD approach with each of the two water models and the DMD approach.

	Turn [%]			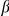 -Strand [%]		
	SPCE	TIP3P	DMD	SPCE	TIP3P	DMD
A 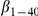	44.4 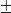 3.7	45.0 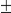 4.0	39.1 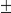 4.2	4.5 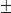 0.6	4.2 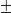 0.5	6.4 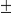 1.0
A 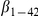	43.9 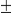 3.5	44.2 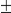 3.5	34.9 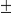 3.5	6.1 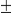 0.7	5.9 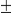 0.8	9.8 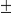 1.3

The error bars correspond to SEM values.

**Table 3:** Average turn, 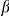-strand, helical, and coil propensities within A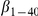 and A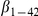 dimers obtained by the all-atom MD approach with each of the two water models and the DMD approach.

	Turn [%]			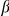 -Strand [%]		
	SPCE	TIP3P	DMD	SPCE	TIP3P	DMD
A 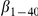	43.5 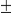 3.6	42.5 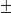 3.6	40.6 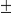 4.1	5.5 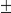 0.8	4.8 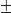 0.6	13.6 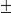 1.6
A 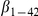	40.1 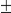 3.2	41.6 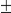 3.1	39.2 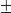 3.7	6.6 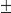 0.8	5.6 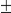 0.7	15.7 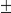 1.9

The error bars correspond to SEM values.

There were two distinct secondary structure differences between the MD and DMD dimers. The MD-derived dimers of both A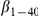 and A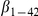 had (i) increased turn propensities in the region A2-F4 and (ii) decreased turn propensities in the C-terminal region V36–V39 relative to the dimers obtained by the DMD approach. Importantly, A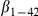 dimers had significantly higher turn propensity in the C-terminal region than A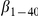 dimers for both water models. A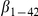 dimers had lower turn propensities at the N-terminal region D7-E12 relative to A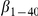 dimers, but the difference was larger for the SPCE water model. In addition, A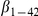 dimers had a lower turn propensity than A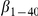 dimers at the N-terminal region A2-F4 but only for the SPCE water model (Fig. S7A).

The 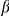-strand propensity per amino acid was significantly decreased for A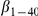 and A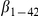 dimers obtained from MD simulations with either of the two water models relative to the DMD-derived dimers (Fig. S8). Despite significantly lower values of the 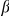-strand propensity in MD, the A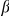 regions with the largest 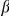-strand propensity remained similar to those predicted by the DMD approach. The 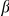-strand maxima between MD and DMD structures mostly coincided. The higher 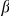-strand contents of DMD dimers was consistent with more structured and compact DMD dimers relative to the fully atomistic MD dimers. Notably, the dimers obtained by MD simulations with the SPCE water model showed slightly increased 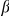-strand propensities than those obtained with the TIP3P water model, but the water model-induced differences were considerably smaller than those between the DMD and MD dimer structures (Fig. S8A and S8B).

The alloform-specific differences in the 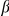-strand propensity in MD dimers were mostly located in the region A30-V40/A42, in which A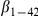 dimers displayed more 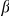-strand structure than A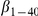 dimers. Here, the region V39-I41 in A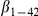 dimers was characterized with 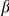-strand structure not present in A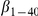 dimers as previously predicted by DMD [19] and consistent with subsequent experimental and computational studies [24]–[26]. The N-terminal region with a nonzero 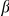-strand propensity in A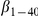 dimers at A2-F4 was shifted to the region E3-R5 in A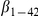 dimers, for both water models. While this structural difference was qualitatively similar to the one observed for DMD structures, it was quantitatively smaller than predicted by DMD.

### Tertiary and quaternary structure of dimers is alloform specific

Tertiary and quaternary structure of A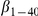 and A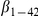 dimers was examined through intra- and intermolecular contact maps defined based upon a proximity between pairs of C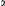 atoms (Fig. S9). Overall, intramolecular contacts were more numerous and stronger than intermolecular contacts for dimers of both alloforms, indicating a stronger tertiary than quaternary structure. Although the two water models resulted in slightly different contact maps, the water model differences were smaller than the differences between the contact maps of A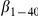 and A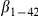 dimers.

A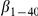 dimers had stronger tertiary contacts than A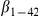 dimers (Fig. S9A–B,E–F). The dominant intramolecular contacts in A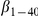 dimers were those between the central hydrophobic cluster (L17-A21) and the mid-hydrophobic region I31-V36, followed by contacts between the central hydrophobic cluster and the N-terminal region A2-F4. A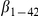 formed stronger intramolecular contacts compared to A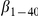 dimers between the central hydrophobic cluster and the C-terminal region V39-A40. These results are qualitatively similar to the tertiary and quaternary structures derived within the DMD approach [19], [21], [58].

The strongest quaternary contacts in A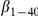 dimers were among the L17-A21 regions, followed by the contacts between the L17-A21 and I31-V36 regions (Fig. S9C–D,G–H). A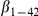 dimers were in comparison characterized with less quaternary contacts among the the L17-A21 regions than A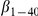 dimers. Instead, the intermolecular contacts involving I31-V36 and the C-terminal region V39-I41 were dominant. This result also qualitatively agrees with the previous DMD-derived results [19], [21], [58].

### Spatial distribution of residues within A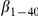 and A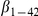 dimers differs

**Figure 5:**
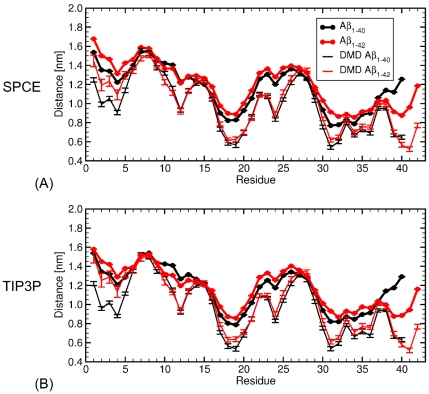
The average distance from the CM of each amino acid residue in A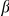 dimers. *The thick black and red curves correspond to the average distances from the CM of amino acids within A* 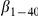 *and A* 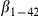 *dimers, respectively, acquired by MD using (A) SPCE and (B) TIP3P water model. The thin black and red curves correspond to the average distances from the CM of amino acids within the corresponding DMD-derived A* 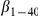 *and A* 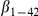 *dimers, respectively. The error bars are SEM values.*

We determined specific differences in the spatial distribution of the residues within all-atom MD A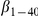 and A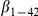 dimers by calculating the distance from the CM for each residue of each of the two peptides comprising a dimer. The data, shown in Fig. 5, indicated an overall increased distance from the CM for A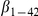 dimers relative to A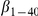 dimers in the N-terminal region D1-R5 and in the region L17-V36, which is strongly hydrophobic. The largest difference involved the C-terminal region, which was on average farther from the CM in A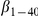 dimers than in A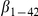 dimers, for both water models. The data for both water models were quite similar, although the SPCE water model resulted in increased distances from the CM. Interestingly, the A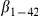 versus A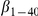 difference in the distance from the CM in the region D1-R5 was larger for the SPCE than for TIP3P water model (Fig. 5).

The DMD approach predicted the distance from the CM, which was smaller than the resulting all-atom MD distances, demonstrating an overall more compact DMD structures. Such a systematic shift towards smaller distances was expected as the DMD approach is combined with a four-bead peptide model, in which the side chain is represented by a single atom/bead and does not account for variable sizes of specific side chains. Qualitatively, however, *the shape* of this distance as a function of the amino acid number followed very well the all-atom MD-derived distances. The exception was the C-terminus, where the DMD distances for both A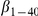 and A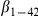 dimers were significantly smaller than all-atom MD distances. This was not surprising considering that the implicit solvent parameter associated with effective electrostatic interactions in the DMD approach was set to zero [21], whereas the negatively charged C-termini in the MD force field were subjected to electrostatic interactions competing with the hydrophobic nature of the C-terminal residues. The differences originating from different water models (SPCE versus TIP3P) were small relative to the differences between the DMD and fully-atomistic dimer structures. Representative fully atomistic MD trajectories of A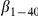 and A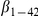 dimers in explicit water (SPCE) are included as animations Movie S1 and Movie S2.

### Distinct residue-specific water density profiles around A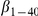 and A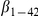 dimers

**Figure 6:**
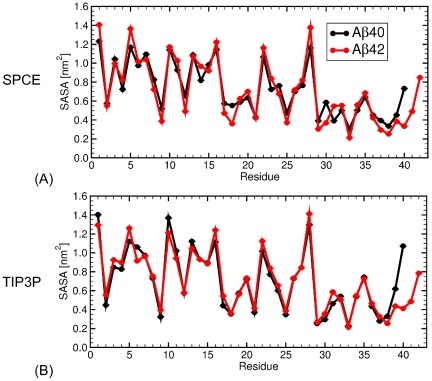
The average SASA per amino acid in A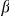 dimers. *The thick black and red curves correspond to SASA for all-atom A* 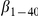 *and A* 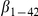 *dimers obtained by MD using the (A) SPCE and (B) TIP3P water model. The error bars are SEM values.*

We calculated the solvent accessible surface area (SASA) per amino acid (Fig. 6). A major difference in SASA between A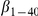 and A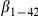 was an increased solvent exposure of the C-terminal region V39–V40 in A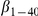 dimers relative to A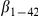 dimers. The two water models resulted in slightly different SASA values for individual residues. The residues that were more exposed to the solvent in A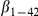 dimers than in A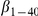 dimers for both water models were the three positively charged residues R5, K16, and K28.

Simulating explicit water molecules interacting with A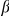 peptides allowed us to calculate the average residue-specific radial distributions of SPCE and TIP3P water molecules around A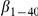 and A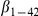 dimers (Figs. S10–S11). These residue-specific water density profiles demonstrated that structural differences between A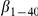 and A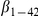 dimers significantly affected the water density profiles at several specific residues along the sequence: (a) D1, R5, Y10, A30, and V40 for the SPCE water model (residues with well-separated non-overlapping SEMs, marked by “**” in Fig. S10) and (b) D1, R5, Y10, and L17 for the TIP3P water model (residues with well-separated non-overlapping SEMs, marked by “**” in Fig. S11). In addition, somewhat alloform-specific water density profiles were observed around residues: (a) E3, V12, H13, Q15, F19, G29, I32, M35, G38, and V39 for the SPCE water model (residues with touching, non-overlapping SEMs, marked by “*” in Fig. S10) and (b) E3, S8, V18, F20, G25, S26, K28, I32, L34, G38-V40 for the TIP3P water model (residues with touching, non-overlapping SEMs, marked by “*” in Fig. S11). Although the primary structure of A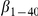 and A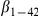 differs at the C-terminus, the three residues that were characterized with distinct water density profiles in both water models (D1, R5, Y10) were located within the N-terminal region of the peptides. In A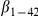 dimers, a reduced number of water molecules in the first solvation shell around D1 and R5 was observed relative to A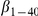 dimers. The situation was reversed for Y10, which was in A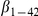 dimers surrounded by a larger number of water molecules in the first solvation shell than in A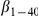 dimers. These analysis showed that the secondary, tertiary, and quaternary structure differences between A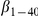 and A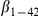 dimers affected the local water density around selected N-terminal residues.

### Salt bridge formation in A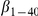 and A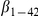 monomers and dimers

**Figure 7:**
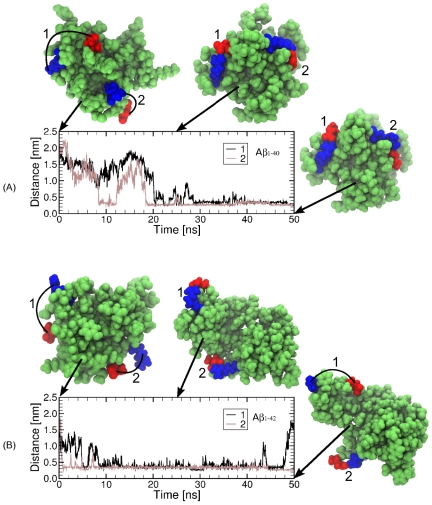
Intrapeptide salt bridge formation between D1 and R5 in A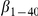 and A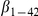 dimers in SPCE water model. *The dimers are colored in green, D1 in red and R5 in blue. The distance between one oxygen of D1 side chain and one nitrogen of R5 side chain for each of the two peptides in a (A) A* 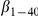 *and (B) A* 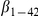 *dimer is shown as a function of simulation time. The D1-R5 salt bridges on each of the two peptides in a dimer are marked as 1 and 2.*

At neutral pH, A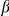 peptides are characterized by three positively charged amino acids: R5, K16 and K28, which can form salt bridges with each of the six negatively charged amino acids: D1, E3, D7, E11, E22 and D23. We calculated all salt bridge propensities in A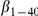 and A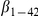 monomers and dimers. The average salt bridge propensities are displayed in Tables 4 and 5. Examples of D1-R5 salt bridge formation and breaking are shown in Fig. 7.

**Table 4:** Salt bridge propensity for A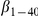 and A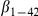 monomers.

	Salt Bridge Propensity [%]			
	Intra peptide			
	A 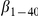		A 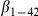	
AA Pair	SPCE	TIP3P	SPCE	TIP3P
R5-D1	8 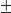 4	27 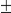 6	36 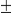 6	37 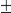 7
R5-E3	17 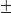 5	24 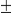 6	28 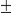 5	32 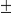 6
R5-D7	19 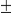 5	11 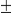 4	4 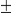 3	6 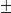 3
R5-E11	11 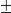 4	18 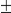 5	3 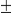 2	14 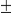 5
R5-E22	8 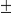 4	12 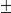 4	7 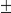 4	12 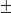 5
R5-D23	2 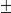 2	4 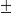 2	7 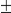 4	2 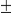 2
**TOTAL R5**	**65** 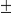 **10**	**96** 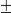 **12**	**85** 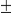 **10**	**103** 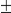 **12**
K16-D1	1 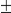 0	3 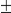 2	5 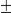 2	2 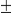 2
K16-E3	2 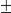 2	2 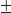 2	2 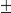 2	5 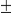 3
K16-D7	3 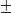 2	3 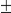 2	3 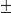 2	10 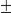 4
K16-E11	7 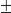 3	7 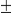 3	9 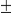 4	8 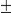 3
K16-E22	3 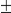 2	6 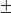 3	3 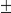 2	4 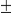 3
K16-D23	5 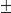 3	2 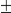 2	4 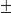 2	5 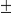 3
**TOTAL K16**	**21** 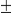 **5**	**23** 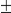 **6**	**26** 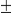 **6**	**34** 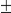 **7**
K28-D1	10 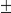 4	9 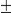 3	0 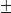 0	6 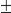 3
K28-E3	6 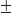 2	3 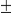 1	3 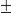 2	9 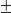 4
K28-D7	0 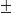 0	3 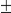 2	4 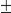 3	2 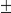 1
K28-E11	1 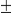 0	5 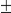 3	5 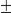 3	6 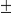 3
K28-E22	12 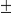 4	12 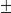 4	10 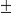 4	15 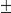 4
K28-D23	9 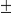 3	14 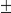 4	8 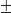 4	14 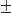 4
**TOTAL K28**	**38** 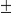 **7**	**46** 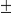 **7**	**30** 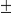 **7**	**52** 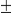 **8**
**Average TOTAL**	**41** 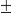 **13**	**55** 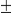 **15**	**47** 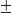 **14**	**63** 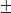 **16**

The error bars correspond to SEM values.

**Table 5:** Salt bridge propensity in A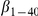 and A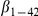 dimers.

	Salt Bridge Propensity [%]											
	Intra peptide				Inter peptide				Total			
	A 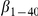		A 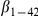		A 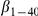		A 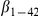		A 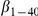		A 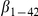	
AA Pair	SPCE	TIP3P	SPCE	TIP3P	SPCE	TIP3P	SPCE	TIP3P	SPCE	TIP3P	SPCE	TIP3P
R5-D1	11 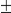 3	9 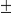 3	27 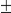 5	26 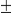 4	2 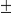 1	1 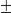 1	3 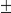 2	0 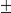 0	13 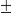 3	10 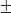 3	30 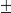 5	27 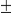 4
R5-E3	18 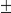 4	32 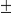 5	27 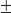 4	36 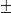 4	3 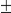 2	0 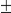 0	2 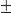 1	1 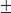 1	21 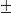 4	32 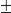 5	28 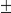 4	36 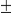 4
R5-D7	14 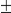 3	12 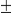 3	13 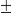 3	16 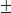 3	1 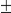 1	0 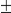 0	1 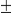 1	0 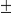 0	15 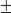 3	12 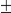 3	14 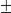 3	16 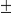 3
R5-E11	8 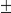 2	6 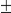 2	3 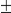 2	6 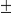 2	1 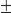 1	5 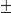 2	2 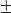 2	1 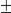 1	9 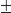 2	11 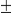 3	6 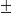 2	8 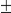 3
R5-E22	7 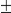 3	8 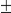 3	6 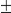 2	5 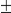 2	3 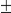 2	5 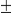 2	2 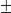 1	5 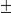 2	10 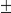 3	13 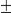 4	8 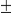 3	10 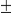 3
R5-D23	4 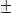 2	3 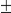 2	1 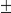 1	1 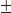 1	4 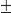 2	2 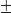 2	2 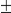 1	3 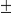 2	8 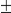 2	5 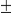 2	3 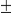 1	4 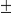 2
**TOTAL R5**	62 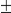 7	70 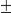 8	77 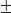 8	90 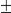 7	14 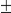 4	13 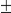 4	12 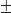 3	10 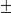 6	**76** 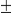 7	**83** 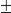 **8**	**89** 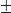 **8**	**101** 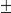 **8**
K16-D1	3 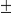 1	7 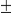 2	1 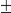 0	2 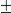 1	7 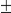 2	2 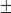 1	1 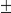 1	2 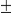 1	10 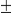 2	9 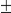 3	1 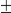 1	4 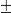 1
K16-E3	2 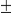 1	4 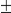 2	2 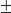 1	4 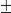 2	6 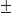 2	2 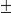 1	1 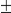 1	3 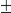 1	8 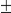 2	6 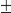 2	4 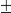 2	6 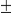 2
K16-D7	4 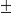 2	4 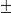 2	3 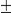 2	1 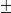 1	1 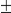 1	0 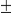 0	3 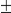 1	1 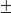 1	5 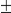 2	4 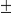 2	6 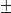 2	2 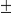 1
K16-E11	4 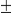 1	9 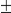 2	5 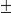 2	4 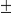 2	1 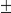 1	0 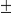 0	2 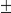 1	4 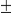 2	5 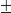 1	9 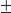 2	7 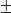 2	9 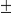 3
K16-E22	1 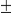 1	3 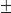 2	1 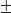 1	2 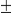 1	1 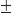 1	2 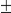 1	0 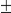 0	2 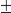 1	2 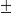 1	5 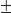 2	1 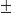 1	4 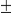 2
K16-D23	1 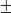 1	2 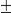 1	1 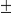 1	1 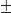 1	3 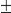 1	1 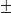 1	3 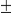 2	3 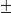 2	4 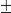 2	4 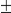 2	5 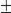 2	4 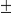 2
**TOTAL K16**	15 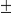 3	29 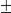 5	13 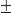 3	14 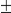 3	19 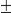 3	7 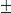 2	10 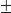 3	15 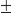 8	**34** 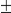 **4**	**37** 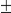 **5**	**24** 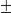 **4**	**29** 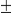 **5**
K28-D1	3 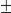 1	4 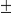 1	4 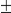 1	5 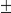 2	5 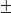 2	3 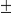 2	1 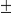 1	1 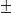 1	7 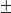 2	7 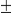 2	5 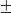 1	6 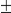 2
K28-E3	1 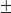 1	3 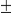 1	1 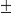 0	2 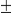 1	3 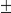 1	5 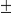 2	1 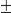 1	0 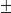 0	4 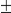 2	8 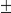 2	2 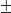 1	2 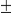 1
K28-D7	3 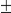 1	4 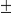 2	2 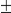 1	2 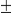 1	2 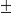 1	0 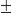 0	1 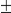 1	3 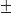 1	6 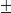 2	5 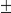 2	4 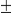 2	4 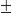 2
K28-E11	2 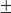 1	2 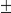 1	2 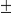 1	2 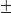 1	0 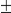 0	1 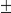 1	3 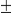 2	3 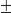 2	2 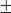 1	4 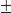 1	5 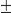 2	5 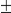 2
K28-E22	9 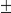 2	11 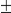 3	4 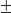 1	8 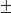 2	0 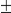 0	5 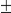 2	0 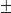 0	4 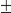 2	9 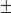 2	15 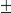 3	4 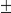 1	13 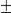 3
K28-D23	15 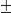 3	17 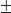 3	5 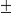 2	9 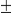 3	2 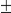 1	3 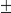 1	1 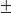 1	3 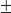 1	17 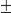 3	20 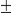 4	6 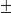 2	13 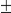 3
**TOTAL K28**	33 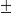 4	41 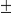 5	18 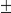 3	28 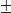 4	12 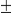 3	17 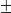 4	7 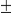 3	14 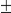 7	**45** 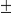 **5**	**59** 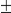 **6**	**26** 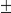 **4**	**43** 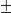 **6**
**Average TOTAL**	**37** 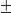 **9**	**47** 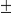 **11**	**36** 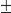 **9**	**44** 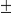 **9**	**15** 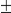 **6**	**12** 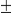 **6**	**10** 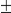 **5**	**13** 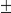 **12**	**52** 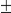 **9**	**60** 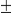 **11**	**46** 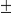 **10**	**58** 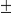 **11**

The error bars correspond to SEM values.

**Figure 8:**
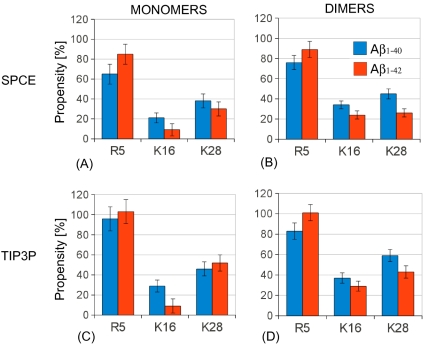
Histograms of salt bridge propensities. *Total salt bridge propensities of the three positively charged amino acids: R5, K16, and K28 in A* 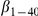 *and A* 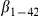 *monomers and dimers are displayed as histograms for each of the two water models.*

The alloform- and water model-specific salt bridge propensities for each of the three positively charged amino acids are shown as histograms in Fig. 8. Of the three positively charged amino acids, R5 was the most involved in salt bridge formation, followed by K28, and K16 had the lowest propensity for salt bridge formation. This result was independent of the water model, alloform, and assembly state. The preference for salt bridge formation involving R5 can be understood by taking into the account the proximity of negatively charged residues D1, E3, and D7. A turn/loop structure centered at G25-S26 enabled the positively charged K28 to be in the proximity to the negatively charged E22 and D23, resulting in E22-K28 and D23-K28 salt bridges. In contrast, the salt bridge counterparts for the positively charged K16 were less obvious as the tertiary and quaternary structure would not favor the proximity of K16 to the nearest negatively charged residues E11, E22, D23.

Interestingly, there was no statistically significant alloform-specific difference in salt bridge propensities for monomers that would simultaneously appear in both water models, although R5 had a tendency to form more salt bridges in A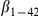 than in A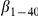 monomers (Table 4). No significant difference in salt bridge formation between A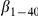 and A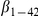 monomers, consistent with our results, was reported in a recent explicit-solvent MD study [46]. Whereas in a recent REMD study of A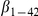 monomers in implicit solvent, the E22-K28 salt bridge was reported to form with a higher propensity than the D23-K28 salt bridge [43], Lin *et al.* showed that the D23-K28 salt bridge occurred more frequently than the E22-K28 salt bridge in both A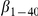 and A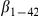 monomers [46], in agreement with our present findings. The MD results of Wise-Scira *et al.* indicated a high salt bridge propensity for the residue R5 in the A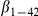 monomer as observed in our simulations (Table 4) [43].

The differences in salt bridge propensities between the two alloforms were larger for dimers. Some salt bridge propensities depended strongly on the water model. For example, A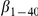 dimers had almost three-fold larger D23-K28 salt bridge propensity than A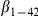 dimers for the SPCE water model. For the TIP3P water model, the difference was smaller (Table 5). For the SPCE but not TIP3P water model, A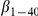 dimers also had an increased E22-K28 salt bridge propensity relative to A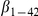 dimers. Among the 15 different salt bridge propensities, those that showed significant alloform-specific differences for both water models were: (i) D1-R5 with a three-times larger propensity in A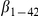 than in A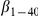 dimers; and (ii) D1-K16 and E3-K28 with a more than two-fold increased propensity in A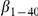 relative to A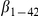 dimers (Table 5). These propensity differences can be understood by considering that the N-terminal region of A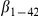 dimers was more exposed to the solvent and interacted less with the other peptide regions than the N-terminal region of A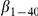 dimers.

## Discussion

A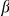 oligomers are central to the pathology of AD yet their structure is experimentally evasive. It is intriguing that a 5% difference in the primary structure between A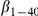 and A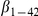 results in distinct *in vitro* oligomerization pathways [15], toxicity [11], [12], and membrane permeability [59]. The first computational study by Urbanc *et al.*, which demonstrated that A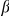 folding and oligomer formation were significantly affected by additional two amino acids in A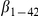, used the DMD approach [19]. In this approach, DMD was coupled with a four-bead protein model with backbone hydrogen bonding [60] and amino acid-specific implicit solvent interactions [61]. Moreover, this DMD approach resulted in distinct folded structures [20] and oligomer size distributions for A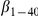, A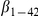, and their Arctic mutants (E22G) [21]. Distinct structural characteristics of A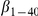 and A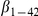 were observed already at the stage of folding. Specifically, the A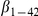 monomer was shown to have an increased 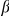-hairpin propensity at the C-terminal region that was absent in the A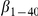 monomer [19]. This observation was corroborated by both experimental [23]–[26] and all-atom MD studies [20], [26], [27], [42], [46]. Moreover, the DMD-derived A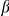 oligomer conformations were qualitatively similar to a recently observed tetramer structure of A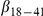 enclosed within the CDR3 loop region of a shark Ig new antigen receptor single variable domain antibody and resolved by x-ray spectroscopy [62].

Based on the successful structural predictions of the DMD approach described above, we hypothesized that the DMD-derived A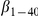 and A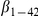 conformations are sufficiently proximate to their fully atomistic counterparts and can be used as viable initial conformations for the all-atom MD study in explicit solvent. Because dimer formation represents a seminal event in A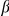 assembly, we here focused on structural characteristics of fully atomistic A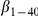 and A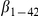 monomers and dimers in explicit solvent. We structurally compared fully atomistic A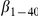 and A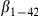 monomers and dimers and quantified their structural differences. Our aim was to elucidate those structural elements that could be associated with distinct toxicities of A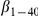 and A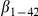 oligomers observed both *in vitro* and *in vivo*.

All-atom MD studies of full-length A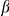 oligomers in explicit solvent are demanding due to a large number of atoms and also because A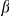 belongs to a family of intrinsically disordered proteins without a well-defined native state in an aqueous solution, resulting in an ensemble of relatively unstructured conformers. On the other hand, in the presence of HFIP or in a membrane-like environment, both A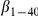 and A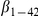 adopt a more ordered helical structure [63], [64]. Recent all-atom MD studies demonstrated the heterogeneous nature of the tertiary structure of the A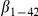 monomer ensemble and the importance of extracting structural characteristics from averaging over the entire conformational ensemble rather than deriving them from a few representative structures [45], [46]. Efficient sampling of the phase space of full-length A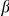 conformations is thus critical for the convergence of structural quantities and is typically addressed by using advanced sampling techniques [26], [39], [44], [49], [50]. To ensure an efficient sampling of the phase space, we selected *a large ensemble of A* 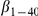 *and A* 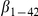 *monomer and dimer structures derived by DMD* [21] as initial conformations for fully atomistic MD simulations using OPLS-AA force field combined with SPCE and TIP3P water models. Comparison of the structural differences between A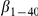 and A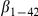 conformations using two water models allowed us to identify those that were robust with respect to the water model.

The resulting all-atom MD structures of A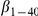 and A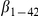 dimers qualitatively resembled that of DMD-derived dimers, with the hydrophobic C-terminal region comprising a core and the N-terminal region exposed to the surface (see Movie S1 and S2). Quantitative comparison revealed that DMD dimers were more compact and displayed more interpeptide contacts than the corresponding MD dimers. This was not surprising, as the four-bead protein model used in the DMD approach reduces all amino acid side chains to a single atom. When fully atomistic side chain templates were superposed onto the four-bead dimer structure, the entire dimer “swelled up” to prevent side chains–backbone or side chain–side chain clashes. Importantly, a key structural difference between A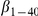 and A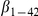 dimers predicted by the DMD approach, the increased solvent exposure of the N-terminal region in A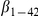 relative to A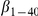 dimers, was *qualitatively preserved* in the present fully atomistic MD-derived dimer structures. This difference was recently hypothesized to be associated with distinct toxicity properties of A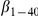 versus A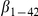 oligomers [29]. The question of why and to which degree coarse-grained peptide models with simplified amino acid description might be successful in predicting assembly structures is still under investigation [53].

We examined specifically the 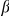-strand propensity per amino acid and the distance from the CM. Overall, the amount of the 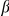-strand structure in MD–derived A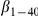 and A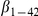 dimers was more than two times *lower* than experimentally measured 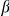-strand content of 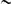
15–25% for A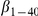 and A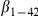 dimers in an aqueous solution [57], [65], [66]. This result was on one hand surprising because the DMD–derived dimers, which were used as initial conformations for all-atom MD, were characterized by the amount of 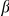-strand comparable to experimental values [21]. According to the experimental [57] and DMD studies [21], the average 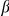-strand should increase upon dimer formation from 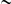
10–20% to 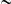
15–25%. Most explicit-solvent MD studies, including the present one result in lower amounts of the 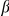-strand content in A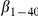 and A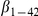 monomers [26], [44]–[46], whereas a larger amounts of 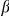-structure were reported for A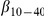 when combined with implicit solvent force field [41]. Although our explicit-solvent MD-derived A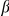 dimers in the SPCE water model had more 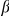-strand structure than those in the TIP3P water model, the difference was not statistically significant. These findings raise a question of an accuracy of the commonly used all-atom force fields and/or explicit water models in MD studies of proteins. Recently, some discrepancies between experimental and computational data on conformational ensembles of the alanine dipeptide and tripeptides have been reported by several groups [67]–[70]. To which extent the amount of the 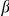-strand structure in all-atom MD-derived A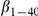 and A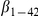 monomers and oligomers depends on the accuracy of the force field and/or the ability of the water model to capture the hydrophobic and hydrophilic effects is still unknown.

We derived the free energy landscapes of A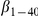 and A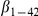 monomers and dimers to characterize structural changes in A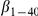 and A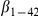 induced by the monomer to dimer transition. The free energy landscapes were derived by characterizing each conformation by the NT-CM distance and SASA of all hydrophobic residues. Upon dimer formation, the minima of both A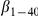 and A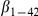 free energy landscapes were significantly shifted towards lower SASA values, demonstrating that dimer formation was driven by effective hydrophobicity. A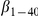 and A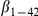 free energy landscapes of dimers (but not monomers) revealed that A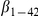 dimers were structurally more diverse than A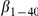 dimers. In addition, the free energy landscape of A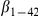 dimers was shifted towards higher SASA values relative to the free energy landscape of A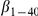 dimers, consistent with a view that a higher solvent exposure of hydrophobic residues correlates with an increased aggregation propensity. The free energy landscape of A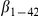 dimers showed a larger number of shallower minima compared to A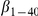 dimers as well as monomers of both alloforms. This result demonstrated that *dimer formation increased the structural disorder in A* 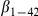 *but not in A* 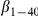 *conformations*.

Our results again demonstrate that considering the exact A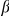 sequence is important for a correct description of A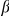 folding and oligomer formation. Takeda and Klimov studied the effect of N-terminal truncation on A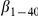 folding by REMD in implicit solvent and demonstrated that the N-terminal region of A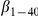 formed contacts with the central hydrophobic cluster [41], [52], [71], in agreement with the DMD predictions for A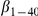 but not for A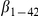 monomers and oligomers [20], [21]. A fully atomistic MD study in explicit solvent by Ball *et al.* showed that A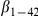 and A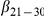 monomers sample quite distinct ensembles of conformations [45]. Similarly, Wise-Scira *et al.* demonstrated that whereas A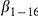 and A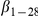 monomers displayed 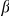-structure at the N-terminus, this feature was diminished in A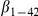 [43]. Explicit solvent MD simulations by Lin *et al.* also showed that the peptide length and single amino acid substitutions affect the A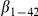 monomer structure [46]. Thus, full-length A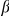 structural characteristics cannot be automatically inferred from the studies of A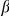 fragments.

Intrapeptide salt bridges were shown to play an important role in stabilizing the A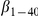 fibril structure [72], [73]. A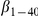 modified by a lactam bridge D23-K28 formed fibrils 1000-fold faster, suggesting that a rate limiting nucleation step was bypassed [74]. To elucidate the role of charged residues in A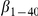 and A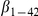 monomer and dimer structures, we analyzed all intra- and interpeptide salt bridge propensities. In our simulations, salt bridge formation and breaking was a dynamic process involving charged residues that were highly solvent accessible. K28 formed salt bridges with all six negatively charged residues, although D23-K28 had the highest propensity, followed by E22-K28. It is known that salt bridges contribute to the stability of proteins only if buried in the interior of the protein structure [75]. Thus, prior to fibril formation the salt bridge D23-K28 needs to undergo the energetically unfavorable burial into the interior of the A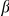 assembly. This burial may represent the rate limiting nucleation step for A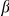 fibril formation, that can be induced by formation of the intrapeptide lactam bridge D23-K28 [74].

Recent *in vitro* studies indicate that A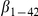 oligomers cause more damage to the negatively charged than electrostatically neutral membranes [76]. Positively charged amino acids R5, K16, and K28 would be natural candidates for interactions with negatively charged membranes. Of the three, our data showed that R5 had the highest propensity for salt bridge formation, followed by K28, whereas K16 had the lowest salt bridge propensity. Interestingly, R5 was more actively forming salt bridges in A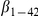 than in A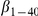 monomers and dimers, consistent with a higher solvent exposure of the N-terminal region in A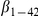, which enabled R5 to form salt bridges with the neighboring D1, E3, and D7. For both water models, A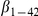 dimers (but not monomers) formed significantly less salt bridges involving K28 than A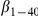 dimers. A smaller propensity for intra- and interpeptide salt bridge formation of K28 and to some extent of K16 in A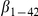 dimers could mean that K28 and K16 are more available to form salt bridges with negatively charged lipid bilayer as supported by a recent experimental reports on oligomer formation and cell culture toxicity [77], [78]. If so, the burial of the salt bridge D23-K28 to the interior of A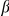 assembly prior to fibril formation might be responsible for a reduced toxicity of A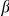 fibrils relative to oligomers.

Alternatively, the ability of positively charged amino acids R5, K16, K28 to form intra- and interpeptide salt bridges may be a reflection of the local structural flexibility of monomers in dimers. If so, then R5, which was shown here to have a higher solvent exposure and a lower number of water molecules in the first solvation shell in A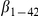 than in A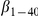 dimers, would be able to more readily interact with negatively charged membrane targets. This conclusion is supported by a recent hypothesis on the critical involvement of the N-terminal region in A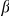 oligomer-mediated toxicity [29] as well as *in vivo* studies, which demonstrated that amino acid substitution within the N-terminal region strongly affected A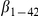-mediated toxicity [79] and that the antibody binding to the N-terminal region (but not to the C-terminal region) of A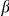 strongly reduced A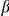-induced toxicity [80].

In summary, our present MD study based on an extensive phase space sampling achieved through combining the DMD-derived A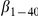 and A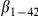 monomer and dimer conformations with all-atom MD in explicit solvent provided new insights into the structural differences between A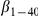 and A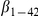 induced by dimer formation that may be relevant to the distinct toxicity properties of the two alloforms. Specifically, our study elucidated the role of structural disorder, water solvation, and salt bridge formation upon A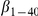 and A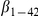 monomer to dimer conversion. The comparison between our fully atomistic and DMD-derived A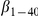 and A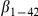 conformations also provides a valuable feedback on the DMD approach, which can be employed to refine its underlying force field and may be of value to other computational approaches based on coarse-grained peptide modeling [81].

## Methods

### All-Atom MD with Explicit Water Model

The MD simulations were performed with the parallel code GROMACS 4.0.7 [82]–[85] and the OPLS-AA [86], [87] force field combined with the SPCE [88] and TIP3P [89] water models. The SPCE water model was chosen because it resulted in the best hydration properties of amino acid analogues among five non-polarizable water models when combined with three commonly used biomolecular force fields, AMBER99, GROMOS 53A6, and OPLS-AA [48]. In addition, the TIP3P water model was selected because the combination of the OPLS-AA force field and TIP3P water model previously resulted in distinct A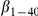 versus A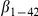 folded structures consistent with experimental data [26]. This strategy allowed us to address the robustness of our structural results with respect to the water model. The cutoff for the Van der Waals interactions of 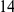 Å was suggested by the creators of GROMACS (see the GROMACS Manual) for optimal results in combination with the OPLS-AA force field. The efficient particle-mesh Ewald (PME) algorithm was used for implementation of long-range electrostatic interactions with the grid dimension of 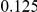 nm and interpolation order of 6. Equations of motion were integrated by the leap-frog method using a time step of 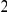 fs.

### Simulation Setup

A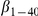 and A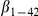 monomer and dimer conformations derived by the DMD approach with the four-bead protein model and implicit solvent residue-specific interactions [21] were converted into the united-atom representation using an in-house software package *protsView*. This process involved the use of united-atom side-chain templates, which replaced the C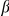 atom of the four-bead conformation. The addition of the side chain templates was followed by an optimization of the contact energy using the Monte Carlo method, separately for backbone and side-chain atoms, to avoid clashes. The final united-atom conformation differed from the initial four-bead conformation by RMSD value smaller than 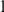 Å. To obtain fully atomistic monomer and dimer conformations, hydrogens were added to the united-atom conformations using Visual Molecular Dynamics (VMD) software package [90].

Each all-atom conformation was inserted into a cubic water box extending 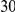 Å from the protein surface in all directions to avoid the interaction of the conformation with its image due to periodic boundary conditions. This procedure resulted in a range of box sizes 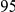
–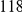 Å. Three (six) Na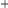 ions were added to neutralize the total charge of the A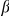 monomer (dimer) in water. All N-termini were positively (NH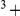) and C-termini negatively (COO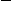) charged. The total number of water molecules was in the range of 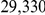
–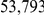 resulting in 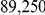
–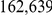 atoms, including the A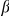 conformation. Each A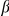-water system was subjected to the energy minimization using the steepest descent algorithm, followed by a 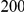 ps equilibration run, during which the heavy atoms were constrained to their initial positions, allowing water molecules to equilibrate around the A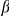 structure. The 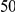 ns production runs were performed in the NPT ensemble. The temperature of 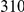 K was maintained by a velocity rescaling thermostat with a stochastic term [91] using a time constant of 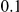 ps and the atmospheric pressure was enforced by the Parrinello-Rahman method [92] using a coupling constant of 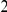 ps. Monomer and dimer conformations were recorded every 50 ps, resulting in 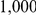 conformations for each 50 ns-long trajectory. The simulations were conducted on *Steele* at Purdue University through the NSF TeraGrid supercomputing resources. For each trajectory, we used 8 cores in parallel, resulting in 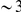 ns of simulation time per day.

### Structural Analysis

#### Contact Number

The contact number was defined as the number of contacts *between* the two peptides within a dimer. Two amino acids belonging to two different peptides were in contact if their respective C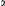 atoms were less than d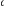 apart. We tested two values of d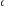, 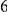 Å and 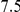 Å, which resulted in the same contact numbers. The contact number provided a measure of the contact surface area that stabilized a dimer structure.

#### Distance from Center of Mass per Amino Acid

To calculate the distance from CM for each amino acid, the CM of each monomer/dimer conformation was first calculated, followed by the calculation of the distance between the position of the C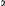 atom of each residue and the CM. For each amino acid, an average value and the SEM was calculated using all A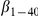 and A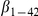 conformations acquired between 20 and 50 ns of each trajectory.

The overall structure of A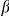 monomers and dimers was quasi-spherical with N-termini exposed to the solvent and buried C-termini. We defined a particular distance of the N-terminal C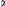 from the CM, the NT-CM distance, as a measure of an effective radius of a dimer conformation.

#### 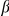-Strand Propensity per Amino Acid

The secondary structure of each amino acid was calculated with the STRIDE program [93] algorithm as implemented within the VMD software package [90]. The average 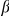 strand propensity per amino acid and the SEM values were calculated by averaging over all A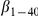 and A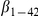 conformations acquired between 20 and 50 ns of each trajectory.

#### Solvent Accessible Surface Area

We calculated the solvent accessible surface area per amino acid (SASA) as implemented within the VMD software package [90]. This calculation used a spherical surface around each of the amino acid atoms, 1.4 Å away from the atom's van der Waals surface. The joint SASA for all atoms in an amino acid was then calculated by taking into account surfaces of all other atoms in the A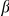 conformation. Amino acids that are buried inside of the conformation (shielded from the solvent) had lower SASA values than amino acids exposed to the solvent.

#### Salt Bridge Propensities

Salt bridges were identified between positively charged amino acids R5, K16 and K28 and negatively charged amino acids D1, E3, E11, E22, and D23 using the VMD software package [90]. We considered a salt bridge formed whenever any of the side-chain nitrogen atoms of a positively charged amino acid was within 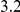 Å distance from any side-chain oxygen atom of a negatively charged amino acid (Fig. 7). The salt bridge propensity was defined as the the total time that the salt bridge was present during 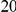
–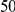 ns of each trajectory divided by the total observation time (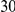 ns per trajectory).

#### Potential of the Mean Force

The potential of the mean force (PMF) was calculated by projecting each monomer or dimer conformation acquired between 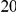
–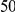 ns of each trajectory onto two selected reaction coordinates. In the phase space of the two reaction coordinates, we created a two-dimensional normalized histogram with 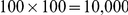 bins and counted the total number of conformations 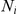 in each bin. The PMF values of each bin were obtained by calculating 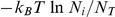, where 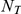 was the total number of conformations. The total number of A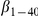 and A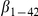 dimer conformations included in each PMF plot is given in parentheses in Table 1.

#### Contact Maps

Two amino acids were considered to be in contact whenever their C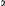 atoms were found below a distance of 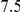 Å as used in the DMD studies [19], [21], [58]. The contact map is the (i,j) matrix with the average number of contacts between two specific amino acids, calculated by averaging over the total number of conformations (see Table 1). The intramolecular contact maps included contacts between the i-th and j-th amino acid that belonged to the same peptide (tertiary contacts). The intermolecular contact maps included contacts between the i-th and j-th amino acid that belonged to different peptides (quaternary contacts). The SEM values are included in all contact map plots.

## Supporting Information

Figure S1
**Temporal evolution of RMSD Values.** *RMSD values for five representative trajectories of each (A,B) A* 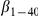 *and (C,D) A* 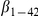 *monomers obtained by MD combined with (A,C) SPCE and (B,D) TIP3P water models.*
(EPS)

Figure S2
**Temporal evolution of RMSD Values.** *RMSD values for five representative trajectories of each (A,B) A* 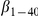 *and (C,D) A* 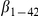 *dimers obtained by MD combined with (A,C) SPCE and (B,D) TIP3P water models.*
(EPS)

Figure S3
**Convergence of the distance from the center of mass of each amino acid residue in A** 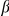 **monomers with simulation time.** *Distance from the center of mass for each C* 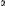 *atom of each amino acid was calculated by averaging over 0–10 ns, 10–30 ns, 30–40 ns, and 40–50 ns of (A,B) A* 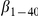 *and (C,D) A* 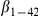 *monomer trajectories, for each (A,C) SPCE and (B,D) TIP3P water model.*
(EPS)

Figure S4
**Convergence of the distance from the center of mass of each amino acid residue in A** 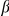 **dimers with simulation time.** *Distance from the center of mass for each C* 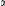 *atom of each amino acid was calculated by averaging over 0–10 ns, 10–30 ns, 30–40 ns, and 40–50 ns of (A,B) A* 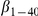 *and (C,D) A* 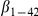 *dimer trajectories, for each (A,C) SPCE and (B,D) TIP3P water model.*
(EPS)

Figure S5
**Convergence of the distance from the center of mass of each amino acid residue in A** 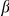 **monomers with the number of trajectories.** *Distance from the center of mass for each C* 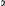 *atom of each amino acid was calculated by averaging over 10, 20, 30, and 40 trajectories of (A,B) A* 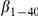 *and (C,D) A* 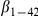 *monomer trajectories between 20 and 50 ns, for each (A,C) SPCE and (B,D) TIP3P water model.*
(EPS)

Figure S6
**Convergence of the distance from the center of mass of each amino acid residue in A** 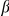 **dimers with the number of trajectories.** *Distance from the center of mass for each C* 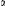 *atom of each amino acid was calculated by averaging over 10, 20, 30, and 40 trajectories of (A,B) A* 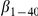 *and (C,D) A* 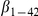 *dimer trajectories between 20 and 50 ns, for each (A,C) SPCE and (B,D) TIP3P water model.*
(EPS)

Figure S7
**The average turn propensity per amino acid in A** 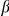 **dimers.** *The thick black and red curves correspond to turn propensities for A* 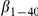 *and A* 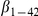 *dimers, respectively, calculated from all-atom MD trajectories between 20 and 50 ns for the (A) SPCE and (B) TIP3P water model. The thin black and red curves correspond to turn propensities of the corresponding DMD-derived A* 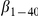 *and A* 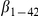 *dimers, respectively. The error bars are SEM values.*
(EPS)

Figure S8
**The average** 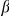 **-strand propensity per amino acid in A** 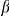 **dimers.** *The thick black and red curves correspond to* 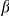 *-strand propensities for A* 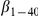 *and A* 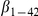 *dimers, respectively, calculated from all-atom MD trajectories between 20 and 50 ns for the (A) SPCE and (B) TIP3P water model. The thin black and red curves correspond to* 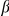 *-strand propensities of the corresponding DMD-derived A* 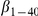 *and A* 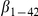 *dimers, respectively. The error bars are SEM values.*
(EPS)

Figure S9
**Average C** 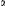 **–C** 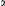 **contact maps in A** 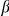 **dimers.** *(A,B,E,F) Intra molecular and (C,D,G,H) inter molecular contact maps for (A,B,C,D) A* 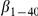 *and (E,F,G,H) A* 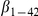 *calculated from MD trajectories with the (A,E,C,G) SPCE and (B,F,D,H) water model. The color scheme to the right of the plots gives the contact probability.*
(EPS)

Figure S10
**Radial distribution function of SPCE water around amino acids in A** 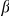 **dimers.** *Distribution of water around each amino acid in A* 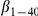 *are shown in black and in A* 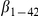 in red. Each amino acid is identified with the one letter code. ** indicates differences between A 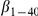 *and A* 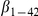 values outside of the SEM and * indicates differences between A 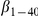 *and A* 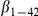 *values for which the SEM are marginal.*
(EPS)

Figure S11
**Radial distribution function of TIP3P water around amino acids in A** 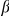 **dimers.** *Distribution of water around each amino acid in A* 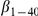 *are shown in black and in A* 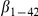 in red. Each amino acid is identified with the one letter code. ** indicates differences between A 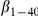 *and A* 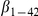 values outside of the SEM and * indicates differences between A 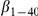 *and A* 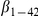 *values for which the SEM are marginal.*
(EPS)

Movie S1
**A** 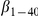 **Dimer Animation.** *Animation of representative fully atomistic A* 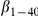 *dimer simulation with OPLS-AA force field and TIP3P water model. In total,* 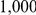 *frames separated by 50 ps were extracted from the 50 ns MD trajectories for each dimer and rendered using the the VMD package. The final animations were encoded at 30 frames/second. Atoms are shown in van der Waals representation. Individual peptides are shown in green and blue, respectively, and N-terminal D1 amino acids are depicted in red.*
(AVI)

Movie S2
**A** 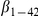 **Dimer Animation.** *Animation of representative fully atomistic A* 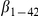 *dimer simulation with OPLS-AA force field and TIP3P water model. In total,* 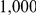 *frames separated by 50 ps were extracted from the 50 ns MD trajectories for each dimer and rendered using the the VMD package. The final animations were encoded at 30 frames/second. Atoms are shown in van der Waals representation. Individual peptides are shown in green and blue, respectively, and N-terminal D1 amino acids are depicted in red.*
(AVI)
